# Updated genome assembly and annotation of *Paenibacillus larvae*, the agent of American foulbrood disease of honey bees

**DOI:** 10.1186/1471-2164-12-450

**Published:** 2011-09-16

**Authors:** Queenie WT Chan, R Scott Cornman, Inanc Birol, Nancy Y Liao, Simon K Chan, T Roderick Docking, Shaun D Jackman, Greg A Taylor, Steven JM Jones, Dirk C de Graaf, Jay D Evans, Leonard J Foster

**Affiliations:** 1 Department of Biochemistry ž Molecular Biology, Centre for High-throughput Biology, University of British Columbia, 2125 East Mall, Vancouver, British Columbia, V6T 1Z4 Canada; 2 Bee Research Laboratory, Agricultural Research Service, United States Department of Agriculture, 10300 Baltimore Avenue, Beltsville, Maryland, 20705, USA; 3 Michael Smith Genome Sciences Centre, British Columbia Cancer Agency, Vancouver, V5Z 4S6, Canada; 4 Ghent University, Laboratory of Zoophysiology, K.L. Ledeganckstraat 35, 9000 Ghent, Belgium and Institute of Marine Sciences Kiel, Düsternbrooker Weg 20, 24105 Kiel, Germany

## Abstract

**Abstract:**

**Background**

As scientists continue to pursue various 'omics-based research, there is a need for high quality data for the most fundamental 'omics of all: genomics. The bacterium *Paenibacillus larvae *is the causative agent of the honey bee disease American foulbrood. If untreated, it can lead to the demise of an entire hive; the highly social nature of bees also leads to easy disease spread, between both individuals and colonies. Biologists have studied this organism since the early 1900s, and a century later, the molecular mechanism of infection remains elusive. Transcriptomics and proteomics, because of their ability to analyze multiple genes and proteins in a high-throughput manner, may be very helpful to its study. However, the power of these methodologies is severely limited without a complete genome; we undertake to address that deficiency here.

**Results:**

We used the Illumina GAIIx platform and conventional Sanger sequencing to generate a 182-fold sequence coverage of the *P. larvae *genome, and assembled the data using ABySS into a total of 388 contigs spanning 4.5 Mbp. Comparative genomics analysis against fully-sequenced soil bacteria *P. JDR2 *and *P. vortex *showed that regions of poor conservation may contain putative virulence factors. We used GLIMMER to predict 3568 gene models, and named them based on homology revealed by BLAST searches; proteases, hemolytic factors, toxins, and antibiotic resistance enzymes were identified in this way. Finally, mass spectrometry was used to provide experimental evidence that at least 35% of the genes are expressed at the protein level.

**Conclusions:**

This update on the genome of *P. larvae *and annotation represents an immense advancement from what we had previously known about this species. We provide here a reliable resource that can be used to elucidate the mechanism of infection, and by extension, more effective methods to control and cure this widespread honey bee disease.

## Background

*Paenibacillus larvae *is a spore-forming, Gram-positive bacterium, studied for the past century due to its ability to cause American foulbrood (AFB), a larval disease of honey bees [1]. The host is most vulnerable during an approximately 48-h window in the life cycle - the early larval stage - where arguably an undeveloped immune system and/or a lack of energy stores result in death. During this period, the oral LD50 ingestion is 8.49 spores [2]; death occurs due to systemic infection after the germinated bacterial spores proliferate in the midgut and then breach the midgut epithelium via a paracellular route [3]. The antibiotics oxytetracycline and tylosin are used both prophylactically and to treat symptoms; however, widespread drug resistance is evident [4] and their registered use is being withdrawn in many countries since residues can show up in honey. Even in susceptible isolates though, it is extremely difficult to completely eliminate from a hive, so without definitive knowledge of the molecular mechanism of pathogenesis, the design of specific treatments is significantly hindered. In 2006, a draft of the *P. larvae *genome was published at an estimated 5-6x coverage [5]; here we extend this coverage and further annotate the genome sequence with a combination of bioinformatics and proteomics.

## Results and Discussion

### Assembly of the *P. larvae *genome

Using the Illumina GAII platform and the ABySS assembler, together with the previously collected Sanger reads [5]; we achieved 182x coverage of the *P. larvae *genome and generated an initial assembly with a contig N50 of 49.6 kb. This statistic describes the contiguity of an assembly, and denotes that 50% of the reconstructed genome is contained in contigs equal to or larger than the given value. Contigs were joined into scaffolds using ABySS and Anchor (version 0.2.7, http://www.bcgsc.ca/platform/bioinfo/software/anchor/releases/0.2.7) (Docking *et al*., in preparation) to achieve a scaffold N50 of 83.2 kb. The largest contig size was 261,601 base pairs. There were 184 contigs with a size less than 1 kb, 136 contigs with length between 1 kb and 10 kb, 57 contigs with length between 10 kb and 100 kb and 11 contigs larger than 100 kb. The total length of the assembled contigs was 4,505,771 base pairs. Assembly statistics are listed in Table 1.

**Table 1:** Summary of the *P. larvae *genome assembly.

Read pairs	8,212,402
Read length	50 bp

Fold coverage	182x

Contig N50	49.6 kb

Scaffold N50	83.2 kb

Number of contigs	388

Total length of assembled contigs	4,505,771 bp

Average GC content per contig	44.04%

Mean contig size	11.6 kb

Largest contig	261,601 bp

**Figure 1:**
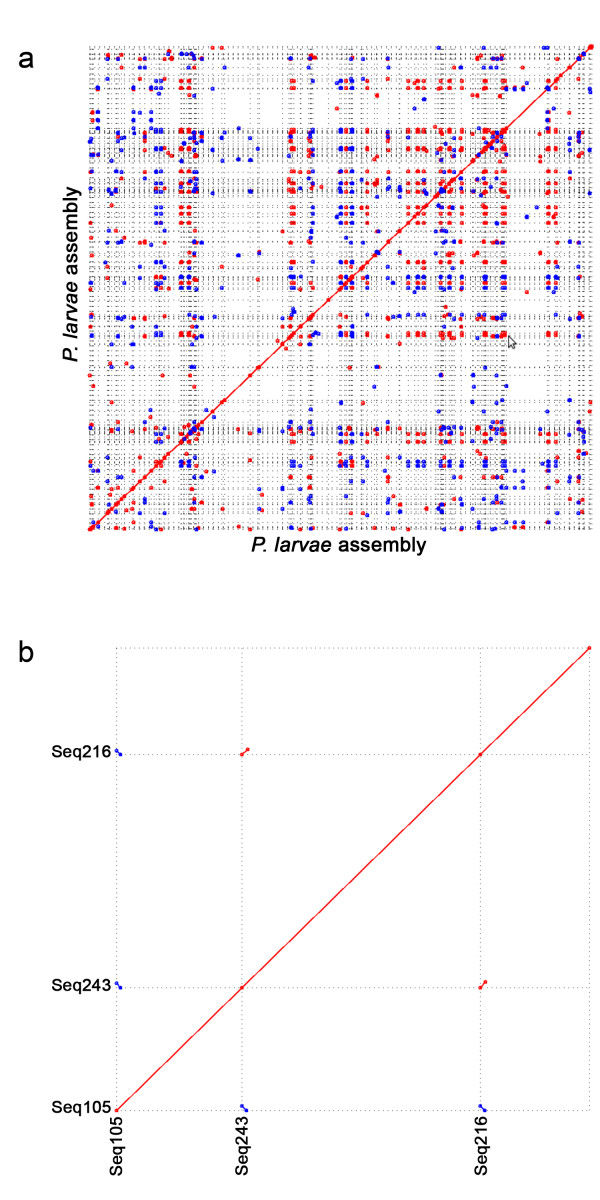
**Self-alignment of 353 *P. larvae *contigs of assembly **ADZY01000000.(a) MUMmer-generated graph of the *P. larvae *contigs aligned with themselves under default parameters. Red dots indicate sense matches and blue dots represent antisense matches. (b) Enlarged view of a sample region to demonstrate the common occurrence of alignments at the ends of contigs.

This Whole Genome Shotgun project has been deposited at DDBJ/EMBL/GenBank under the accession ADZY00000000, with the latest version being ADZY02000000. Genome annotation and downstream analysis described below is based on the first version, ADZY01000000. Despite high sequence coverage, the assembly was relatively fragmented (Figure 1a). This fragmentation appears to be due primarily to long genomic repeats that could not be bridged by our sequencing strategy, as indicated by a preponderance of repetitive sequence occurring at contig ends (example shown in Figure 1b). Many of these repeats are similar to known bacterial insertion sequences and exceed 1 kb in length (data not shown). As a result, the majority of contigs were 1-10 kb in length.

**Figure 2:**
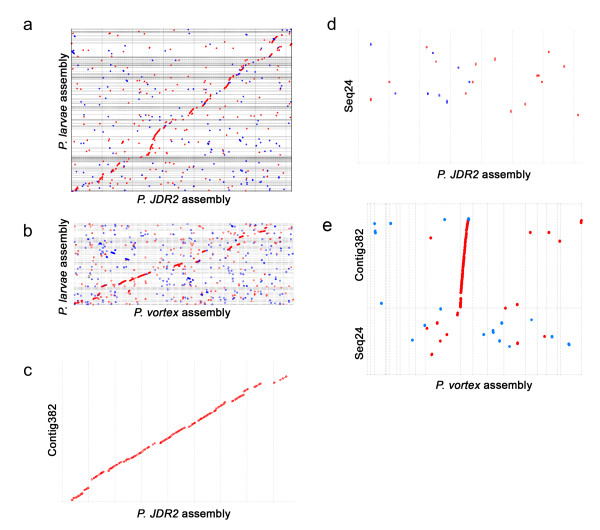
**Comparative genomics for aligning *P. larvae *contigs.MUMmer-generated dot-plot showing *P. larvae *contigs (y-axis) aligned with the fully-sequenced genomes of (a) *P. JDR2 *and (b) *P. vortex *(x-axes)**. Proteins sequences were used for matching. Red dots indicate same-direction matches and blue dots represent antisense matches. Magnified views of regions from (a) exemplify *P. larvae *contigs that are (c) highly conserved and (d) poorly conserved with *P. JDR2*; the same contigs are also compared in *P. vortex *(e).

Given the fragmented nature of the assembly and the potential contribution of transposable elements to chromosome re-arrangements, we used tBLASTx and MUMmer to investigate the level of synteny between *P. larvae *and the congeneric soil bacterium *P. JDR2*. Although the completely sequenced *P. JDR2 *genome is 7.2 Mbp, substantially larger than our assembly (4.4 Mbp), the species share ~93% identity at 16S ribosomal loci. Figure 2a shows that, despite the difference in assembly size, gene-level synteny is generally conserved with *P. JDR2 *across the entirety of many *P. larvae *contigs. Some regions have excellent conservation (example in Figure 2b). This suggests that *P. JDR2 *can be a useful reference for comparative genomics and to order *P. larvae *contigs for genome finishing. Divergent regions between the two genomes are also of interest because they may harbor species-specific genes that are ecologically important in soil and beehive environments, respectively. For example, contig Seq24, which lacks synteny with *P. JDR-2 *(Figure 2c), contains two gene regions not present elsewhere in *P. JDR-2 *that are potential virulence factors (Table 2). Interesting, the same contig was also poorly conserved in another bacterium *P. vortex *(Figure 2d), thereby strengthening the claim that genes in this contig are unique to *P. larvae*. *P. vortex *is a pattern-forming soil bacterium with a 6.4 Mbp genome [6], so as expected, its overall level of synteny with *P. larvae *is quite high (Figure 2e), though slightly lower than observed when compared against *P. JDR-2*.

**Table 2:** Potential *P. larvae *virulence factors in genomic regions not found in *P. JDR2*.

ORF	Strand	Start	Stop	Best match E-value	Best match taxon	Best match accession	Description
EFX43964	minus	1200	2189	1.00E-024	*Bacillus subtilis*	NP_389716.2	plipastatin synthetase

EFX43965	minus	2207	7786	1.00E-110	*Bacillus subtilis*	NP_389598.3	polyketide synthase of type I

EFX43966	minus	7790	12370	1.00E-107	*Streptococcus mutans*	BAH88686.1	NcpA protein

EFX43967	minus	12556	15906	0	*Bacillus subtilis*	NP_389714.1	plipastatin synthetase

EFX43968	minus	15902	19897	0	*Bacillus subtilis*	NP_388230.2	surfactin synthetase

EFX43969	minus	19920	25211	0	*Bacillus subtilis*	NP_389714.1	plipastatin synthetase

EFX43970	minus	25232	34267	0	*Bacillus subtilis*	NP_388230.2	surfactin synthetase

EFX43971	minus	34309	42072	0	*Bacillus subtilis*	NP_389598.3	polyketide synthase of type I

EFX44032	minus	110449	111363	3.00E-090	*Bacillus subtilis*	NP_388265.1	putative iron-siderophore ABC transporter (binding lipoprotein)

EFX44033	minus	111493	112248	1.00E-097	*Bacillus subtilis*	NP_388264.1	putative iron-siderophore ABC transporter (ATP-binding protein)

EFX44034	minus	112245	113180	9.00E-094	*Bacillus subtilis*	NP_388263.1	putative iron-siderophore ABC transporter (permease)

EFX44035	minus	113188	114057	8.00E-095	*Bacillus subtilis*	NP_388262.1	putative iron-siderophore ABC transporter (permease)

Within the regions unique to *P. larvae*, one includes several open reading frames (ORFs) with strong homology to the synthetases of the antibiotic plipastatin, which inhibits phospholipase A2 [7] and surfactin [8], which possess hemolytic activity [9]. The region also contains putative type I polyketide synthetases whose products are secondary metabolites, some of which have antibiotic, immunosuppresant, or toxic effects [10]. The second region encodes homologs of a putative iron-siderophore ABC transporter, which enables iron uptake from the medium, an important process for many bacterial infections [11].

### Annotation of putative *P. larvae *proteins

Using GLIMMER to predict genes from the *P. larvae *assembly and BLAST [12] searches against *Bacillus *or *Streptococcus *to provide annotation information (Additional File 1), we identified 3,568 gene models (final list in Additional File 2). Using shotgun proteomics methods to analyze *P. larvae *lysates, we identified 1262 proteins (1% false discovery rate), thus confirming the expression of 35% of the predicted proteins. Details regarding the identities of these proteins can be found in Additional File 3. As a major aim of this project is to find potential virulent factors and unique genes for this niche-specific bacterium, we describe below the genome content of *P. larvae *for several pathways considered to be important in this regard. The putative functions of the proteins were predicted by searching for matches against the Conserved Domain Database (CDD), and the complete results are tabulated in Additional File 4.

### Flagellar system

**Figure 3:**
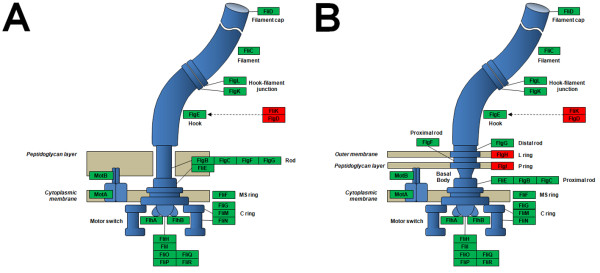
**Flagellar proteins**. Distribution of flagellar proteins (excluding chemotaxis proteins) among flagellated Gram-positive (A) and Gram-negative (B) bacteria. The proteins encoded by the *P. larvae *genome are boxed in green (the ones that are lacking: in red).

*P. larvae *is a flagellated bacterium and 41 genes associated with this system are detected in this assembly (Table 3), based on comparisons to two other fully-sequenced organisms. This group includes motor/switch, flagellar rings, rod hook and filament proteins, along with proteins involved in regulation and as chaperones. When compared against the *Bacillus subtilis *(Figure 3a, based on [13]) and *Escherichia coli *(Figure 3b, based on [14]) flagella, *P. larvae *has orthologs for nearly all of the genes. Those that are missing, such as the L and P ring proteins FlgH and FlgI in the outer membrane of the Gram-negative *E. coli*, are not necessary for the Gram-positive *P. larvae *since it only has one membrane [15]. However, it appears to be missing some players needed for assembly of the flagellar hook, a structure that acts both as a joint and motor for each individual flagellum. The hook itself, made of monomers, requires the monomer scaffolding protein FlgD [16]; this was not found in the *P. larvae *genome. Also missing is FliK, which acts essentially as a checkpoint for the flagellum's correct length prior to export. This may mean that this species has evolved ways to proceed with hook assembly despite their omission, or the enzymes were simply not detected by our current methods; as with many negative results, however, our inability to detect such genes does not imply *P. larvae *is completely incapable of quality control functions. Besides the flagellar structure, another important aspect is the control of its movement. Directionality is largely dictated by the Che gene family and methyl-accepting chemotaxis proteins [17], which can also be found in the *P. larvae *genome. The base of the flagellum consists of assembly and regulatory components, which are made of Fli and Mot gene families in *E. coli *[18]. Again, orthologs for most of these can be seen in *P. larvae*.

**Table 3:** Putative *P. larvae *flagellar proteins

	Gene	Protein, functional information from CDD	*P. larvae *protein accession
Two component system chemotaxis proteins	cheA	CheA, sensor kinase [EC:2.7.13.3]	EFX46098; EFX46097
	
	cheW	CheW, purine-binding protein	EFX46097
	
	cheX	CheX	Not found
	
	cheC	CheC	EFX44110; EFX46096
	
	cheD	CheD [EC:3.5.1.44]	EFX46095
	
	cheR	CheR, methyltransferase [EC:2.1.1.80]	EFX46532
	
	cheB	CheB, response regulator [EC:3.1.1.61]	EFX46099
	
	cheY	CheY, response regulator	EFX46498; EFX46108
	
	cheZ	CheZ	EFX46498
	
	cheV	CheV, response regulator	EFX46097; EFX46099

Methyl-accepting chemotaxis proteins	mcp	methyl-accepting chemotaxis protein	EFX44725; EFX44051; EFX45552; EFX46097
	
	tsr	McpI, serine sensor receptor	EFX44051; EFX44725
	
	tar	McpII, aspartate sensor receptor	EFX44725; EFX46877
	
	trg	McpIII, ribose and galactose sensor receptor	EFX44725
	
	tap	McpIV, peptide sensor receptor	Not found
	
	aer	Aerotaxis receptor	EFX44725; EFX46877
	
	hemAT	haem-based aerotactic transducer	EFX44725; EFX46877

Type-III secretion proteins for flagellar assembly	fliH	FliH	EFX46029
	
	fliI	FliI, flagellum-specific ATP synthase [EC:3.6.3.14]	EFX46028
	
	fliZ, fliO	FliO/FliZ	EFX46107
	
	fliP	FliP, flagellar biosynthetic protein	EFX46106
	
	fliQ	FliQ, flagellar biosynthetic protein	EFX46105
	
	fliR	FliR, flagellar biosynthetic protein	EFX46104
	
	flhA	FlhA, flagellar biosynthesis protein	EFX46102
	
	flhB	FlhB, flagellar biosynthetic protein	EFX46103
	
	flhE	FlhE	Not found
	
	flhF	FlhF, flagellar biosynthetic protein	EFX46101

Motor/Switch	motA	MotA, chemotaxis protein	EFX45489
	
	motB	MotB, chemotaxis protein	EFX45490

C-ring	fliG	FliG, flagellar motor switch protein	EFX46030
	
	fliM	FliM, flagellar motor switch protein	EFX46110
	
	fliNY, fliN	FliN/FliY, flagellar motor switch protein	EFX46109

M, S, P and L rings	fliF	FliF, flagellar M-ring protein	EFX46031
	
	flgI	FlgI, flagellar P-ring protein precursor	not found
	
	flgA	FlgA, flagella basal body P-ring formation protein	not found
	
	flgH	FlgH, flagellar L-ring protein precursor	not found
	
	fliF	FliF, flagellar M-ring protein	EFX46031
	
	flgA	FlgA, flagella basal body P-ring formation protein	not found
	
	flgH	FlgH, flagellar L-ring protein precursor	not found

Rod, hook, and filament	flgB	FlgB, flagellar basal-body rod protein	EFX46034
	
	flgC	FlgC, flagellar basal-body rod protein	EFX46033
	
	flgD	FlgD, flagellar basal-body rod modification protein	not found
	
	flgF	FlgF, flagellar basal-body rod protein	EFX46113; EFX45368
	
	flgG	FlgG, flagellar basal-body rod protein	EFX46113; EFX45367
	
	flgJ	FlgJ, flagellar protein	not found
	
	flgE	FlgE, flagellar hook protein	EFX46113
	
	fliE	FliE, flagellar hook-basal body complex protein	EFX46032
	
	flgK	FlgK, flagellar hook-associated protein 1	EFX46010
	
	flgL	FlgL, flagellar hook-associated protein 3	EFX46011
	
	fliD	FliD, flagellar hook-associated protein 2	EFX46016
	
	fliK	FliK, flagellar hook-length control protein	not found
	
	fliL	FliL, flagellar protein	EFX46111
	
	fliC	Flagellin	EFX46015
	
	flaF	FlaF, flagellar protein	EFX46015
	
	flaG	FlaG, flagellar protein	not found
	
	flbA	FlbA, flagellar protein	EFX46102
	
	flbB	FlbB, flagellar protein	not found
	
	flbC	FlbC, flagellar protein	not found
	
	flbD	FlbD, flagellar protein	not found
	
	flbT	FlbT, flagellar protein	not found

Regulation	flhC	FlhC, flagellar transcriptional activator	not found
	
	flhD	FlhD, flagellar transcriptional activator	not found
	
	fliA	FliA, RNA polymerase sigma factor for flagellar operon	EFX44501;EFX46093; EFX43745; EFX44502
	
	flgM	FlgM, negative regulator of flagellin synthesis	not found

Chaperones	flgN	FlgN, flagella synthesis protein	not found
	
	fliJ	FliJ, flagellar protein	EFX46027
	
	fliS	FliS, flagellar protein	EFX46017
	
	fliT	FliT, flagellar protein	not found
	
	fliW	FliW, flagellar assembly factor	EFX46013
	
	fliY	FliY, cystine transport system substrate-binding protein	EFX46109
	
	fliZ	FliZ	EFX46107

Archaeal flagellar proteins	flaA-A, flaA	FlaA, archaeal flagellin	EFX46015
	
	flaB-A, flaB	FlaB, archaeal flagellin	EFX46015
	
	flaC-A, flaC	FlaC	EFX46015
	
	flaD-A, flaD	FlaD	EFX46015
	
	flaE-A, flaE	FlaE	EFX46113
	
	flaF-A, flaF	FlaF	EFX46015
	
	flaG-A, flaG	FlaG	not found
	
	flaH-A, flaH	FlaH	not found
	
	flaI-A, flaI	FlaI	not found
	
	flaJ-A, flaJ	FlaJ	not found
	
	flaK-A, flaK	FlaK, archaeal preflagellin peptidase	not found

### Toxins

Our search for *P. larvae *genes that can encode toxins returned three matches to sixteen proteins (Table 4). A domain of the *Clostridum perfringens *epsilon toxin (pfam03318) was observed in EFX46729 and EFX45732, which forms pores on host cells [19] leading to cell death. *P. larvae *appear to possess classic binary toxins: the first factor is a membrane component that makes the host cell permeable to a second factor - the enzymatic component. Seven proteins matched the *Clostridial *binary toxin B domain (pfam03495), the membrane component. We also saw that four proteins matched the VIP2 domain (cd00233), which is the enzymatic component of a *Bacillus *toxin capable of ADP ribosylation on actin. In both the *Clostridial *and *Bacillus *toxins, the associated partners of these binary toxin-pairs were not observed.

**Table 4:** Putative *P. larvae *toxins.

Protein (E-value)	Domain, functional information from CDD
EFX46729 (8.00E-20), EFX45732 (1.00E-18)	ETX_MTX2 [pfam03318]: Clostridium epsilon toxin ETX/Bacillus mosquitocidal toxin MTX2

EFX43878 (4.00E-32), EFX45096 (3.00E-22), EFX46664 (1.00E-17), EFX46330 (1.00E-10)	VIP2 [cd00233]: A member of the vegetative insecticidal protein family. An actin-ADP-ribosylating toxin. A binary toxin.

EFX43879 (2.00E-102),	Binary_toxB [pfam03495]: Clostridial binary toxin B/anthrax toxin PA. A binary toxin.
EFX45058 (1.00E-83),	
EFX45095 (2.00E-48),	
EFX44748 (3.00E-37),	
EFX46332 (2.00E-29),	
EFX44749 (6.00E-19),	
EFX46331 (1.00E-16),	
EFX44747 (4.00E-6),	
EFX44951 (6.00E-5),	
EFX46847 (6.00E-5),	

*the listed match is not the one with the lowest E-value; it is displayed because it is the most informative

### Hemolysins

In the mid to late stage of the *P. larvae *infection cycle, bacteria circulate in bee hemolymph which contains hemocytes. Hemolysins are a class of bacterial toxins with lytic activity against blood cells. Four hemolysin domains were matched by proteins from the *P. larvae *genome. (Table 5). Three proteins contained regions that were highly similar to the hemolytic domain TylC (COG1253) and concomitantly to other domains such as CBS_pair_CorC_HylC_assoc and DUF21 (data not shown), implying that these are very similar proteins. EFX46836 was matched to the hlyIII domain, an integral membrane protein from *Bacillus cereus *which forms channels on host cells [20]. EFX45686 appears to be related to hemolysin TlyA (TIGR00478) with an indirect pore-forming role. One protein (EFX45724) showed similarity to DUF37 with unpublished claims of hemolysin activity in *Aeromonas hydrophila*.

**Table 5:** Proteins with domains related to hemolysins or cytolytic activity.

Protein (E-value)	Domain, functional information from CDD
EFX44255 (8.00E-69), EFX44544 (8.00E-72), EFX46152 (3.00E-69)	TylC [COG1253]: Hemolysins and related proteins

EFX46836 (2.00E-23)	hlyIII [pfam03006]: channel-forming cytolysin

EFX45724 (1.00E-22)	DUF37 [cl00506]: found in various bacteria, a member from Aeromonas hydrophila has been found to have hemolytic activity (unpublished).

EFX45686 (2E-56*)	tly [TIGR00478]: hemolysin TlyA family protein; at least two members of this protein family have been characterized indirectly as pore-forming hemolysins.

*the listed match is not the one with the lowest E-value; it is displayed because it is the most informative

### Proteases

The occurrence of proteases in larval scales (remains of *P. larvae*-infected bee larvae) has already been well documented [21]. A more recent study hints on a definite possibility that one or more such proteins can act as the virulence factor [22]. Since it has been shown that vegetative rods residing in the host midgut can traverse the epithelium by a paracellular route, *P. larvae *may generate proteolytic activity that can disrupt epithelial cell-to-cell junctions. We searched the proteins for domains with this function, and after excluding ones with roles that are not directly implicated in pathogenesis (e.g. signal peptidase, SOS response for DNA repair), we list the matches in Table 6. Based on the information provided by the CDD, several proteins may conceivably serve as virulence factors. The Clp protease complex, in particular, has been linked to virulence factor production in Gram-positive bacteria, as well as degradation of misfolded proteins [23,24]. Proteins EFX45851, EFX44792 may be capable of cleaving the transmembrane regions of substrate proteins (matching domains cd06161, cd06158). Given that tight junctions between epithelial cells are mainly composed of occludin and claudin proteins, both of which have transmembrane domains [25], they may be specific substrates for *P. larvae *proteases. These points, however, are merely speculative, and the many proteases in Table 6 await experimental evidence to prove whether or not they actually contribute a real role in virulence.

**Table 6:** Putative *P. larvae *based on matches to conserved domains.

Protein (E-value)	Domain, functional information from CDD
EFX44344 (2E-37)	AmpS [COG2309]: Leucyl aminopeptidase (aminopeptidase T)

EFX45451 (6E-13)	APP-like [cd01092]: Prolidase and Aminopeptidase P

EFX47002 (0)	clpC [CHL00095]: Clp protease ATP binding subunit

EFX46058 (4E-5)	ClpP [COG0740]: Protease subunit of ATP-dependent Clp proteases

EFX44161 (1E-85), EFX45289 (1E-82)	clpP [PRK00277]: Clp protease proteolytic subunit

EFX45288 (7E-170)	clpX [PRK05342]: Clp protease ATP-binding subunit

EFX45551 (7E-82),	COG0826 [COG0826]: Collagenase and related proteases
EFX45550 (4E-48),	
EFX45481 (3E-19),	
EFX44736 (1E-28)	

EFX44399 (9E-8*)	COG1310 [COG1310]: Predicted metal-dependent protease of the PAD1/JAB1 superfamily

EFX46420 (8E-149)	COG2317 [COG2317]: Zn-dependent carboxypeptidase

EFX44401 (9E-45)	COG2738 [COG2738]: Predicted Zn-dependent protease

EFX44533 (2E-9), EFX45796 (2E-9)	COG2856 [COG2856]: Predicted Zn peptidase

EFX46883 (2E-31)	COG3740 [COG3740]: Phage head maturation protease

EFX45850 (6E-11)	COG4942 [COG4942]: Membrane-bound metallopeptidase

EFX46250 (1E-34), EFX44926 (2E-64)	degP_htrA_DO [TIGR02037]: periplasmic serine protease, Do/DeqQ family

EFX47184 (5E-45)	DegQ [COG0265]: Trypsin-like serine proteases, typically periplasmic

EFX46573 (5E-91),	FtsH_fam [TIGR01241]: ATP-dependent metalloprotease FtsH
EFX44535 (5E-20*),	
EFX44172 (3E-17*)	

EFX47022 (0)	HflB [COG0465]: ATP-dependent Zn proteases

EFX45319 (1E-36*), EFX44073 (5E-8*)	HflC [COG0330]: membrane protease subunits, stomatin/prohibitin homologs

EFX46036 (4E-157)	HslU [COG1220]: ATP-dependent protease HslVU (ClpYQ), ATPase subunit

EFX46037 (8E-65)	HslV [COG5405]: ATP-dependent protease HslVU (ClpYQ), peptidase subunit

EFX43995 (2E-97),	LasB [COG3227]: Zinc metalloprotease (elastase)
EFX45639 (1E-78),	
EFX45177 (9E-58)	

EFX45284 (0)	Lon [COG0466]: ATP-dependent Lon protease

EFX43687 (8E-131)	M3_fam_3 [TIGR02290]: oligoendopeptidase, pepF/M3 family

EFX44444 (2E-74)	M14_Endopeptidase_I [cd06229]: Peptidase M14-like domain of Gamma-D-glutamyl-L-diamino acid endopeptidase 1

EFX44181 (2E-45)	MrcA [COG5009]: Membrane carboxypeptidase/penicillin-binding protein

EFX45553 (4E-10*),	MrcB [COG0744]: Membrane carboxypeptidase (penicillin-binding protein)
EFX46407 (3E-97*),	
EFX44298 (3E-75*),	
EFX46175 (2E-122*),	
EFX44515 (1E-12*),	
EFX44516 (4E-11*)	

EFX45371 (7E-9), EFX45028 (2E-7), EFX45493 (4E-25)	NlpD [COG0739]: Membrane proteins related to metalloendopeptidases

EFX44427 (8E-144)	pepF [TIGR00181]: oligoendopeptidase F

EFX46173 (4E-9)	PepN [COG0308]: Aminopeptidase N

EFX45105 (1E-66)	PepP [COG0006]: Xaa-Pro aminopeptidase

EFX47190 (3E-18)	Peptidase_M23 [pfam01551]: Peptidase family M23

EFX46425 (8E-126)	Peptidase_M6 [pfam05547]: Immune inhibitor A peptidase M6, a metallopeptidase from Bacillus thuringiensis that cleaves host antibacterial proteins

EFX47085 (4E-29)	Peptidase_M9_N [pfam08453]: Peptidase family M9 N-terminal

EFX46343 (8E-6*)	Peptidase_S24 [pfam00717]: Peptidase S24-like

EFX43847 (9E-13)	Peptidase_S41_CPP [cd07560]: C-terminal processing peptidase; serine protease family S41

EFX43943 (3E-27*)	Peptidase_S66 [pfam02016]: LD-carboxypeptidase; this cleaves the bond between an L- and a D-amino acid

EFX45480 (6E-38), EFX45479 (2E-8)	Peptidase_U32 [pfam01136]: Peptidase family U32

EFX43960 (8E-78), EFX45983 (2E-59)	Peptidases_S8_11 [cd04843]: Peptidase S8 family domain, uncharacterized subfamily 11

EFX46806 (8E-36)	Peptidases_S8_Lantibiotic_specific_protease [cd07482]: Peptidase S8 family domain in Lantiobiotic (lanthionine-containing antibiotics) specific proteases

EFX44534 (5E-26), EFX45795 (1E-20*), EFX44174 (8E-25)	Peptidases_S8_Subtilisin_like_2 [cd04847]: Peptidase S8 family domain in Subtilisin-like proteins

EFX45938 (8E-46), EFX45020 (3E-62), EFX46180 (2E-50), EFX45070 (2E-20)	Peptidases_S8_Subtilisin_subset [cd07477]: Peptidase S8 family domain in Subtilisin proteins

EFX44839 (5E-67), EFX44567 (5E-47), EFX44567 (4E-55)	Peptidases_S8_Thermitase_like [cd07484]: Peptidase S8 family domain in Thermitase-like proteins

EFX47149 (7E-172)	peptidase-T [TIGR01882]: peptidase T

EFX45664 (5E-87)	PepT-like [TIGR01883]: peptidase T-like protein

EFX46054 (6E-33), EFX46055 (2E-7), EFX46066 (2E-67)	PqqL [COG0612]: Predicted Zn-dependent peptidases

EFX45492 (3E-73)	prc [TIGR00225]: C-terminal peptidase (prc)

EFX44827 (3E-16*)	PRK00016 [PRK00016]: putative metalloprotease

EFX44779 (1E-110)	PRK02858 [PRK02858]: germination protease

EFX45078 (5E-118)	PRK07318 [PRK07318]: dipeptidase PepV

EFX44480 (4E-11*)	PRK08554 [PRK08554]: peptidase

EFX45711 (2E-73)	PRK09795 [PRK09795]: aminopeptidase

EFX45631 (4E-24*), EFX44757 (1E-22*)	PRK13914 [PRK13914]: invasion associated secreted endopeptidase

EFX44490 (3E-22*)	PRK14791 [PRK14791]: lipoprotein signal peptidase

EFX46689 (1E-42)	S14_ClpP_1 [cd07016]: Caseinolytic protease (ClpP)

EFX44792 (2E-28)	S2P-M50_like_1 [cd06158]: Uncharacterized homologs of Site-2 protease (S2P), zinc metalloproteases (MEROPS family M50) which cleave transmembrane domains of substrate proteins, regulating intramembrane proteolysis (RIP) of diverse signal transduction mechanisms

EFX44503 (2E-42)	spore_II_GA [TIGR02854]: sigma-E processing peptidase SpoIIGA

EFX45851 (1E-12)	S2P-M50_SpoIVFB [cd06161]: SpoIVFB Site-2 protease (S2P), a zinc metalloprotease (MEROPS family M50B), regulates intramembrane proteolysis (RIP)

EFX47001 (2E-8*)	spore_lon_C[TIGR02903]: ATP-dependent protease, Lon family

EFX45285 (0)	spore_lonB [TIGR02902]: ATP-dependent protease LonB

EFX46081 (7E-45)	TIGR00054 [TIGR00054]: RIP metalloprotease RseP

EFX44693 (1E-61)	trio_M42_hydro [TIGR03106]: hydrolase, peptidase M42 family

*the listed match is not the one with the lowest E-value; it is displayed because it is the most informative

### Antibiotic resistance

*P. larvae *infections have been treated with tetracycline for decades. It has been shown that resistance against this antibiotic can be conferred by the pMA67 plasmid [4]. More recently, the macrolide tylosin has now been registered as a new antibiotic in the U.S. for use when tetracycline is no longer effective [26]. Since antibiotic resistance is inevitable [27], new therapeutics will eventually be required. Realizing that *P. larvae *appears to encode a plethora of drug efflux proteins (Table 7) may help in the selection of useful drugs.

**Table 7:** Putative P. larvae antibiotic resistance proteins based on matches to conserved domains

Protein (E-value)	Domain, functional information from CDD
EFX43850 (3E-37), EFX47127 (3E-26), EFX43889 (2E-44),	ABC_BcrA_bacitracin_resist [cd03268]: The BcrA subfamily represents ABC transporters involved in peptide antibiotic resistance; bacitracin is an antibiotic produced by B. licheniformis and B. subtilis with potent antibiotic activity against gram-positive bacteria
EFX46602 (1E-57),	
EFX44842 (2E-50),	
EFX46272 (4E-41),	
EFX45233 (9E-52)	

EFX46614 (4E-25),	ABC_DR_subfamily_A [cd03230]: This family of ATP-binding proteins belongs to a multisubunit transporter involved in drug resistance (BcrA and DrrA), nodulation, lipid transport, and lantibiotic immunity
EFX44699 (4E-16),	

EFX45065 (1E-50)	ABC_DrrA [cd03265]: DrrA is the ATP-binding protein component of a bacterial exporter complex that confers resistance to the antibiotics daunorubicin and doxorubicin

EFX46465 (1E-55),	ABC_drug_resistance_like [cd03264]: ABC-type multidrug transport system, ATPase component
EFX45467 (3E-61),	
EFX45475 (2E-48*)	

EFX43892 (1E-42),	ABC_putative_ATPase [cd03269]: This subfamily is involved in drug resistance, nodulation, lipid transport, and bacteriocin and lantibiotic immunity
EFX46872 (1E-35),	
EFX47099 (4E-20*),	
EFX45045 (1E-71*)	

EFX46236 (3E-6*)	ABCC_MRP_Like [cd03228]: The MRP (Mutidrug Resistance Protein)-like transporters are involved in drug, peptide, and lipid export; they belong to the subfamily C of the ATP-binding cassette (ABC) superfamily of transport proteins.

EFX44443 (3E-26)	ACR_tran [pfam00873]: AcrB/AcrD/AcrF family. Members of this family are integral membrane proteins. Some are involved in drug resistance

EFX44702 (4E-33),	CcmA [COG1131]: ABC-type multidrug transport system, ATPase component
EFX46006 (5E-34),	
EFX44595 (4E-16),	
EFX43880 (8E-54),	
EFX47052 (7E-40),	
PL1_2983 (1E-33),	
EFX46146 (6E-40),	
EFX45253 (3E-22),	
EFX45810 (9E-18),	
EFX45945 (1E-18),	
EFX47082 (9E-21*),	
EFX44360 (3E-15*),	
EFX46805 (6E-30*),	
EFX45180 (5E-27*)	

EFX43881 (3E-4*)	COG0842 [COG0842]: ABC-type multidrug transport system, permease component

EFX44713 (1E-52)	COG2409 [COG2409]: Predicted drug exporters of the RND superfamily

EFX47096 (4E-18)	COG4152 [COG4152]: ABC-type uncharacterized transport system, ATPase component

EFX46793 (6E-34)	COG4586 [COG4586]: ABC-type uncharacterized transport system, ATPase component

EFX46612 (2E-58),	drrA [TIGR01188]: daunorubicin resistance ABC transporter ATP-binding subunit
EFX43786 (8E-63),	
EFX45226 (4E-54),	
EFX44634 (3E-36*)	

EFX43785 (4E-5)	drrB [TIGR01247]: daunorubicin resistance ABC transporter membrane protein; the protein associated with effux of the drug, daunorubicin

EFX44398 (8E-11), EFX47139 (1E-59)	efflux_Bcr_CflA [TIGR00710]: drug resistance transporter, Bcr/CflA subfamily

EFX46169 (4E-40),	efflux_EmrB [TIGR00711]: drug resistance transporter, EmrB/QacA subfamily
EFX44387 (7E-37),	
EFX44446 (8E-09),	
EFX46307(5E-24),	
EFX46126 (1E-53),	
EFX44956 (3E-9*)	

EFX46708 (3E-14)	emrE [PRK09541]: multidrug efflux protein

EFX44177 (2E-4*)	Glyoxalase [pfam00903]: Glyoxalase/Bleomycin resistance protein/Dioxygenase superfamily

EFX45543 (4E-10),	HTH_MARR [smart00347]: helix_turn_helix multiple antibiotic resistance protein
EFX43861 (8E-11),	
EFX46148 (6E-10),	
EFX46170 (4E-12),	
EFX44712 (3E-12),	
EFX45783 (3E-6*),	
EFX46306 (1E-8*),	
EFX46227 (3E-4*),	
EFX44716 (5E-9*)	

EFX45439 (2E-48)	Lant_dehyd_C [pfam04738]: Lantibiotic dehydratase, C terminus

EFX45438 (3E-31)	LanC [cd04793]: LanC is the cyclase enzyme of the lanthionine synthetase. Lanthinoine is a lantibiotic, a unique class of peptide antibiotics

EFX46802 (4E-77*), EFX46803 (4E-12*)	LcnDR2 [COG4403]: Lantibiotic modifying enzyme

EFX45784 (1E-84),	MdlB [COG1132]: ABC-type multidrug transport system; ATPase and permease components
EFX45333 (6E-107),	
EFX44415 (7E-47*),	
EFX44416 (5E-44*),	
EFX45434 (1E-26*)	

EFX45348 (3E-36)	NorM [COG0534]: Na+-driven multidrug efflux pump

EFX45532 (1E-114)	PRK01766 [PRK01766]: multidrug efflux protein

EFX46249 (6E-16)	PRK03545 [PRK03545]: sugar efflux transporter

EFX46262 (2E-15), EFX44433 (8E-82)	PRK09874 [PRK09874]: drug efflux system protein MdtG

EFX45329 (5E-21)	PRK10054 [PRK10054]: putative MFS-type transporter YdeE

EFX47111 (5E-4*)	PRK10504 [PRK10504]: multidrug efflux system protein MdtE

EFX45115 (3E-58*)	PRK10522 [PRK10522]: multidrug transporter membrane component/ATP-binding component

EFX47080 (1E-129)	PRK10789 [PRK10789]: putative multidrug transporter membrane\ATP-binding components

EFX47079 (5E-98)	PRK10790 [PRK10790]: putative multidrug transporter membrane\ATP-binding components

EFX45606 (2E-13)	PRK11102 [PRK11102]: bicyclomycin/multidrug efflux system

EFX46841 (4E-19), EFX46842 (3E-13)	PRK11431 [PRK11431]: quaternary ammonium compound-resistance protein SugE

EFX43805 (8E-7*)	PRK11646 [PRK11646]: multidrug resistance protein MdtH

EFX47100 (4E-6)	RND_mfp [TIGR01730]: RND family efflux transporter, MFP subunit

*the listed match is not the one with the lowest E-value; it is displayed because it is the most informative

It is known that the bee midgut contains a vast array of bacteria, some of which can inhibit *P. larvae *growth *in vitro *[28-30], presumably by the production of lantibiotics. In our search we were able to find a number of different drug and lantibiotic efflux pumps and modifying enzymes (Table 7), and it is likely that these proteins are enlisted by *P. larvae *during interactions with co-occurring antagonistic bacteria.

## Conclusions

In this article we report an update on the *P. larvae *genome to 182-fold coverage, and estimated the ordering of contigs based on comparison against the *P. JDR2 *genome. We predicted more than 3500 genes and provided these with functional annotation. Of great interest are enzymes that allow the bacterium to traverse the midgut epithelium after ingestion, a process which can contribute to its virulence. Proteases have been thought of as a key factor [22], and this is supported by the large repertoire of such enzymes which we have seen from the predicted gene products. Damage to the host is likely the result of mixed effects from the hemolysins and toxins that can be encoded by *P. larvae*. Its ability to survive in the host by evading the immune system, as well as protecting itself against other gut bacteria, is likely made possible due to the antibiotic proteins that may be expressed.

This improved version of the *P. larvae *genome and the more detailed annotations will be tremendously useful for improving our understanding of this species; for example, in designing primers to clone genes, or mutate them by site-directed mutagenesis. Especially for the field of proteomics, which often relies heavily on a database of protein sequences to make identifications, the current genome update will be a very powerful tool. Computer-based modeling techniques can be employed to predict the structure of virulence factors, which can guide drug design. Ultimately, the genome will pave the way to more effective prevention; or better yet, a cure for AFB. Given the bee population is under severe strain from the widely publicized Colony Collapse Disorder, attributable to multiple biotic and abiotic factors, it is important to continually improve our knowledge of the key threats that burden worldwide bee health.

## Methods

### Preparation of genomic DNA

*P. larvae *strain BRL-230010, stored at -80°C was cultured for 5 d in a shaking incubator at 35°C in 25 mL of media. This media contained 1% Yeast Extract (Difco), 1% soluble starch which was prepared in 10 mM KH_2_PO_4 _and adjusted to pH 6.6 with KOH (Recipe #4 from [31]). Genomic DNA was extracted using the DNeasy kit (Qiagen), following the manufacturer's protocol for Gram-positive bacteria.

### Genome sequencing

8,212,402 2 × 50 bp paired-end reads with a mean fragment size of 182 bp were sequenced at the Genome Sciences Centre, Vancouver, Canada using the Illumina GAIIx platform as per manufacturer's instructions. This yielded approximately 182-fold coverage of the estimated genome size. In addition, 24,768 Sanger capillary-derived sequences were generated at the Human Genome Sequencing Centre, Baylor College of Medicine, using the ABI3730 sequencing platform. This provided an additional 3-fold coverage. Raw Sanger sequences were aligned against the UniVec database (NCBI http://www.ncbi.nlm.nih.gov) using BLAST. Those Sanger sequences that had good BLAST hits (e-value < 1e-10) were filtered from the dataset. Then, vector sequences were trimmed from the raw Sanger reads using cross_match (Cross_match http://www.phrap.org/) against the UniVec database. We then aligned all the Illumina reads to the Sanger sequences using MAQ version 0.7.1 [32]. Due to observed Honey Bee DNA contamination arising within the Sanger sequences, only sequences where at least 30% of the length aligned with Illumina reads were retained.

In the first stage of the assembly process, the Illumina reads were assembled into contigs using ABySS version 1.2.7 [33]. Assemblies were attempted using several different values for the ABySS sequence overlap parameter *k *(which determines the k-mer length used to construct the assembly graph), with the best assembly found at *k = *37. Other modified ABySS parameter values were *s *= 100, *n *= 10, and *d *= 50. All other ABySS parameters were set to their default values. Following the initial assembly, contigs were joined into scaffolds by aligning the long-insert Sanger sequence data to the assembly, and merged using the scaffolding module of ABySS (Jackman *et al*., in preparation). Where possible, scaffold gaps were filled, and contigs were extended, using the local reassembly tool Anchor (version 0.2.7, http://www.bcgsc.ca/platform/bioinfo/software/anchor/releases/0.2.7) (Docking *et al*., in preparation).

### Genome annotation

ORFs were predicted with GLIMMER [34] using the long-ORF training script provided with the software package. ORFs were annotated as predicted proteins if they have at least 100 amino acids or at least 50 with a BLAST hit at a threshold of E = 10^-5 ^to *Bacillus *or *Streptococcus *(Additional File 1). Annotation terms were transferred from FASTA headers where appropriate, and in accordance with NCBI Prokaryotic Genome Submission Guidelines (full list of proteins in Additional File 2).

Genomic dot-plot comparisons were made with the program MUMmer [35]. To produce Figure 2, *P. larvae *contigs were ordered manually based on BLAST hit coordinates to *P. JDR2 *and *P. vortex *as well as the graphical output of MUMmer alignments.

The genome annotation as described above was accomplished using the first version of the assembly from this sequencing (ADZY01000000), which had a total N50 of 56,120 base pairs, a total length of 4,352,422, and a mean contig size of 12,329 bp. When we repeated this analysis with the latest assembly, all but 53 of the 3,568 gene models were identical. Of the 53, most were strong partial hits. All downstream analysis was done using ADZY01000000.

### Shotgun proteomics of P. larvae lysates

*P. larvae *culture was prepared as above. Bacteria were collected by centrifugation for 5 minutes at 16100 relative centrifugal force and the pellet was washed twice in PBS. For an in-gel digestion, the bacteria were lysed in 100 μl of 4× LDS and heated for 15 minutes at 90°C, passed 5 times through a 26 G needle fitted with a syringe before heating for another 10 minutes at 90°C. Lysis was observed under the microscope. Protein concentration was estimated by Coomassie Plus staining, and 200 μg of protein was divided among all 10 wells of a 2-12% NuPAGE precast MES mini gel set up according to the manufacturer's instructions and the in-gel digestion proceeded essentially as described in [36]. For in-solution digestions, bacteria was lysed by either 100 μl of 6 M urea, 2 M thiourea, 20 mM Tris-HCl, pH 8.0 or 0.2 μl RapiGest in 100 μl NH_4_HCO_3 _pH 8 and boiled for 6 minutes. Bacteria from both samples were lysed by the needle and syringe method as above. In a third sample, bacterial surface proteins were stripped by adding 100 μL of 100 mM glycine pH 2 and proteins precipitated by ethanol before being denatured using urea/thiourea as above. Proteins were reduced, alkylated, digested with LysC (urea sample only) and trypsin and purified as described in [36]. Proteins from all three samples, as well as gel fragments, were fractionated by strong cation exchange STAGE tips [37]. Peptides were subjected to liquid chromatography-tandem mass spectrometry on an LTQ-Orbitrap and FT-ICR (Thermo) according to procedures described in [36]. The raw data were searched against the new *P. larvae *protein set derived as above using MaxQuant (v1.1.1.25) [38], considering up to two missed cleavages, Cys carbamidomethylation as a fixed modification and Met oxidation and N-terminal acetylation as variable modifications. The overall mass accuracy of the data set was 0.99 ppm and the false discovery rate, at the protein level, was set to 1%.

### Bioinformatic annotation of predicted proteins

Predicted proteins were BLAST-searched against selected amino acid sequences using the stand-alone BLAST-software package [12]. For the defined targets, i.e. 'flagellar system', 'toxins', 'hemolysins' and 'proteases', query files of representative protein sequences in FASTA format were composed by key word searching the 'protein' database of the National Center of Biotechnology Information (NCBI) at http://www.ncbi.nlm.nih.gov/. The outcome of each BLASTp-search was verified by cross searching the found hits on the online BLASTp tool (NCBI) and by an in depth, manual comparison of their conserved domain architecture and sequence identity. To expand our search for relevant domains, we used BLASTp to search the CDD [39] which contain domain sequences from various sources (Pfam v 24, COG v1, SMART v5.1, Entrez protein clusters database, TIGRFAM v9.0, NCBI, Entrez multi-model superfamilies), with the maximum E-value set at 10^-3^. Where there are matches to more than one domain, we only highlight the one with the lowest E-value in the manuscript. Full results are shown in Additional File 4.

## Authors' contributions

QWTC did all of the wet-bench work, coordinated the whole project and led the writing of the manuscript. SJMJ oversaw the sequencing and assembly. IB, NYL, SKC, SDJ, TRD and GAT conducted all the genome sequencing and assembly. RSC conducted contig ordering. QWTC, RSC, and DCdG did the function annotation and analysis. JDE and LJF had the initial idea for this project and guided the analyses. All authors helped to write the manuscript. All authors have read and approved the final manuscript.

## Supplementary Material

Additional file 1 **Annotation of *P. larvae *proteins**. This table provides annotation information for predicted *P. larvae *proteins found using BLAST searches against *Bacillus *or *Streptococcus*. Click here for file

Additional file 2 **Predicted gene models for the *P. larvae *genome**. The information here is generated from GLIMMER, which was used on the *P. larvae *genome to predicted gene models. Click here for file

Additional file 3 **Mass spectrometry-sequenced *P. larvae *peptides**. Shown here are shotgun proteomics sequenced peptides of *P. larvae *lysates, which serve as experimental evidence for predicted proteins. Click here for file

Additional file 4 **Functional annotation of predicted *P. larvae *proteins based on matches to conserved domains**. Protein sequences of *P. larvae *(column A) were searched against the Conserved Domain Database by BLAST. All matches with an Expect Value (column F) of less than 10-3 are reported here (column B, C), with the hit having the lowest Expect Value marked in column G. A brief description of the domains' function is shown in column D and E. Additional qualifiers such as percent identity, match length, score are found in columns H to P. Click here for file
